# Progress in Development of Functional Biological and Synthetic Blood Products to Augment Transfusable Blood Supply in Operational Medicine

**DOI:** 10.3390/bioengineering12030256

**Published:** 2025-03-04

**Authors:** Armando Estrada, Orion Furmanski, George J. Klarmann, Nathan Scheidt, Vincent B. Ho

**Affiliations:** 1 4D Bio^3^ Center for Biotechnology, Department of Radiology and Bioengineering, Uniformed Services University of the Health Sciences, Rockville, MD 20850, USA; armando.estrada.ctr@usuhs.edu (A.E.III);; 2 The Geneva Foundation, Bethesda, MD 20817, USA; 3 The United States Air Force Academy, USAF Academy, CO 80840, USA

**Keywords:** erythrocyte, red blood cell, transfusion, hemoglobin, synthetic, erythropoiesis, bioreactor

## Abstract

A reliable, accessible, and high-quality blood supply is critical for the sustainment of any healthcare system. World events such as the COVID-19 pandemic have proven that maintaining the supply of blood presents a logistical challenge. The current blood supply is overseen by extensive donor programs around the world. In the United States, as in other countries, the need for blood has increased, with a decline in blood donations and increasing exclusions for blood donor qualification. While there is a need to improve blood donation participation, there is also need for new alternatives to traditional donation to ensure readiness to treat hemorrhagic shock common in the setting of trauma, as often occurs during a natural disaster or conflict. These operational medicine scenarios require significant blood availability which may tax the current blood supply chain. Aside from a walking blood bank (WBB) model for blood collection in suboptimal conditions, researchers have proposed alternatives for blood that include the manufacturing of blood from stem cell sources. Other alternatives include synthetic liquids that can carry oxygen such as Perfluoro-Chemicals (PFCs) and hemoglobin-based oxygen-carrying systems (HBCOs). Here, we review some of these alternatives to the traditional donor blood model. Researchers now have the technology that makes it feasible to develop blood alternatives that one day may supplement and help alleviate the limitations in blood supply.

## 1. Introduction

The United States sees on average one transfusion every 2 s. Currently, 36,000 units of blood are transfused daily in the United States alone; this is made possible by the work of organizations such as the American Red Cross to organize and recruit donors to meet those demands [1,2]. Even with millions of donations per year, the blood supply is limited, and the COVID-19 pandemic further exacerbated these limitations within this logistical chain [3]. The lack of blood caused difficult decisions such as cancelling non-urgent surgeries and cutting transfusion volumes to avert blood shortages [4]. The COVID-19 pandemic led to the closure of many medical clinics and cancellation of most blood donation drives to minimize patient exposures, which resulted in a marked drop in blood donation. This highlights the fragility of the current blood bank model that relies exclusively on donation. As a result, the supply of blood was at its lowest point in a decade, resulting in the American Red Cross declaring a national emergency [3,5,6].

Access to a dependable and safe supply of blood is a requirement for any operational medical mission [7]. This necessitates a replenishable and continuous supply of blood products, which presents a logistical concern for any disaster relief effort or military deployment [7,8]. The American Red Cross as well as the United States Military have developed sophisticated networks to ensure a ready supply of blood, notably for the transfusion needs of patients injured during a natural disaster or military conflict [9,10]. In the case of natural disasters such as a major earthquake or a large-scale combat operation (also known as LSCO), the availability of blood to transfuse into injured and bleeding patients is critical to ensure their survival. In both cases, issues with logistical support and key infrastructure such as power, available healthcare workers, and transportation may limit the ability to quickly replenish the blood supply or the required medical supplies required for screening and transfusing blood [6,7]. The network to deliver blood to frontline first responders and troops may also be compromised due to other external factors which include the survivability of transport vehicles such as ambulances or restricted access due to ambient exposures such as radiation or chemical toxins that may exist in the environment [11].

In the United States, the network to deliver the blood supply consists of a large system of primarily nongovernmental but federally regulated, independent, and nonprofit organizations which has proven to be effective for over half a century [1,12,13]. However, this system, when tested, has been stretched to its limits and is in danger of being overburdened [2,14]. As experienced during the COVID-19 pandemic, the United States Department of Health and Human services, in their report “Adequacy of the National Blood Supply”, emphasized the fragility of the US high-quality blood supply, which is very tenuous and is significantly challenged during natural disasters or other emergency situations such as conflicts. This report recommended additional research to seek alternatives to traditional blood donation to compensate for the current trends of an aging donor population and declining number of qualified donors [15]. In this review, we will discuss the potential for bioengineered transfusable blood alternatives to fill current and future needs in operational medicine.

## 2. Traditional Whole Blood Donations

Healthcare systems worldwide rely on an ample blood supply to treat patients with hemorrhagic shock that commonly occurs following severe trauma such as a hurricane, a motor vehicle accident, or a gunshot wound. This vital part of the healthcare system is maintained by the donation of over one hundred million units of blood each year around the world [16]. Blood donations are primarily driven by charitable foundations such as the American Red Cross who promotes awareness and participation through social media campaigns, blood donation drives, and working with healthcare professionals [17,18]. Although these strategies have vastly improved the public’s awareness and increased donations, the dramatic blood supply shortfall experienced during the global COVID-19 pandemic demonstrate the dire need to maintain a sufficient blood supply [17].

Initially, blood transfusions were utilized in point-of-care facilities as the ability to prevent blood clotting limited blood storage capacity [19]. The identification of ABO blood groups made it possible to transfer blood accurately and safely between individuals with the same blood type with reduced risk of rejection [20]. The technology to store and transfer blood was developed in response to the unprecedented requirements of the First World War [21]. At the onset of the war, it was not possible to meet the requirement for blood on such a massive scale and soldiers succumbed to otherwise non-fatal wounds due to limited access to transfusable blood [22]. The war brought numerous healthcare innovations in blood storage methods, which were aided by discoveries that citrate inhibited clotting while glucose increased the viability and usability of stored blood for multiple weeks under refrigerated conditions [23]. A crucial step in blood transfusion procedures was achieved when the US Army Medical Corps collected Group O blood modified with glucose and stored it before the battle of Cambrai in 1917. This innovation heralded the dawn of a new era that shifted the “donor to recipient” period in medicine to an “indirect” blood transfusion in which blood could be “banked” for prolonged periods of time [24]. This innovation resulted in a more feasible and valuable medical intervention that is practiced to the present day. Today, an estimated 100 million units of blood donated every year are stored in safe conditions and are used to treat trauma and diseases such as anemia [16]. However, donated blood is primarily reserved for acute-blood loss and medical procedures. Hospitals require adequate blood supply on hand for patients undergoing surgical procedures who may develop intraoperative or postoperative bleeding and thus require urgent blood transfusion [25]. Similarly, the acceptance of trauma patients in emergency departments requires an available blood supply or else new admissions of trauma patients may be diverted to other hospitals having adequate blood supplies [26].

Recruitment of healthy blood donors is a primary bottleneck in maintaining a transfusable blood supply. In the United States, the overall national donor base has declined 3% per year over the past decade, and the average age of remaining active donors is steadily increasing. Less than 5% of the US population donated blood in 2018 and only an estimated 15–23% are “eligible” to donate [15]. A growing list of exclusion criteria restricts the number of qualified blood donors [27]. The American Association of Blood Banks (AABB) established certain criteria that potential donors must meet to donate blood. To be eligible, potential donors must be at least 16 years old, weigh no less than 110 pounds (50kg), not have unregulated hypertension, diabetes, anemia, and not be ill at the time of donation [18]. Table 1 demonstrates several of the common criteria that will result in potential donors being disqualified from giving blood:

The need for blood is as pressing as ever for the military as hemorrhage remains the leading preventable cause of death on the battlefield [28]. Access to blood is such a key component to military planners that US Central Command has ordered the storage of type-O “universal donor” whole blood to be distributable to its units starting in 2023 [29]. Operational medicine presents an additional set of challenges beyond those faced by the civilian system in terms of maintaining a transfusable blood supply. Mobile facilities and specialized equipment must be rapidly deployed to the operational theater that, in most cases, has limited resources. Blood donations are overseen by specialized staff, such as phlebotomists and registered nurses, who are trained to monitor donors’ vital signs for potential adverse events [30,31]. Operational conditions pose logistical challenges in terms of transportation, limited refrigerated blood storage facilities, electric power requirements, blood expiration dates, and limited on-site donors [29]. In response to these constraints, the military has implemented a “buddy” system as a source of blood commonly known as the “walking blood bank” (WBB) [29,32].

## 3. Walking Blood Bank

Certain events such as large-scale military operations, natural disasters, or global pandemics can strain supply chains. Under such austere conditions, readily transfusable blood is collected from a WBB [33,34]. The WBB has been in use since World War 1 during the early part of the 20th century [35]. The Department of Defense Joint Trauma System and the Committee on Tactical Combat Casualty Care developed guidelines for the implementation of a WBB [36]. The program pre-screens and registers servicemembers according to their blood type. Qualified donors are of low titer type-O whole blood (LTOWB) and pass the following tests: low antibody titer (<256 anti-A and -B) and negative tests for various diseases such as HIV. Qualified donors are utilized for on-site blood donations and “walking buddy” blood transfusions [36,37].

During combat operations, death from hemorrhagic shock is most likely to occur between the first 6 to 12 h [38]. Prehospital Trauma Registry data obtained between 2003 and 2019 revealed that the average combat casualty resuscitation of wounded servicemen and women received 6 to 13 units of RBCs, depending of the severity of the wounds [19,39,40]. During the military campaigns in Iraq and Afghanistan, increased survival was observed in patients who received blood transfusions early and closer to the site of the where the injury occurred [40]. Lifesaving blood was donated by the WBB, where an on-site blood collection successfully improved outcomes [32].

WBB were first developed for military operations, and it was critical to determine whether blood donations given in the field hindered military operation due to any donor symptoms. Early investigations revealed that people who had donated blood displayed reduced hemoglobin levels and, in turn, reduced oxygen uptake. More recent studies now indicate that military personnel who have donated a minimum of 450 mL of blood displayed no difference in performance in both controlled and in combat-like environments [32]. These data therefore suggest that WBB is not only vital for military operations but also safe for donors in combat operations to help save the lives of their comrades [2]. Both routine blood donation and WBB are a vital part for both civilian emergencies and military operations. However, large-scale combat operations or significant natural disasters will require larger supplies of blood and solely relying on donated blood and using WBB will be challenging. Therefore, additional alternative approaches such as blood manufacturing and blood substitutes must be considered and further developed [15].

## 4. Manufactured Blood—Erythropoiesis In Vitro

Erythropoiesis is the production of mature red blood cells (RBCs) from multipotent hematopoietic stem cells (HSCs) [41]. RBC development in vivo occurs in the bone marrow in complex microenvironmental niches. These so-called “erythroblastic islands” contain supportive cells that provide paracrine signaling cues, cell-to-cell interactions, and mechanical forces that contribute to RBC production. Adult human bone marrow is capable of producing upwards of 2 million RBCs per second [42]. Advances in bioengineered bone marrow-like cell culture technology may have the potential to complement shortfalls in donor blood availability under austere or emergent conditions. However, ex vivo generation of RBCs for therapeutic application in comparable quantities to normal bone marrow has proven to be a monumental challenge.

**Figure 1:**
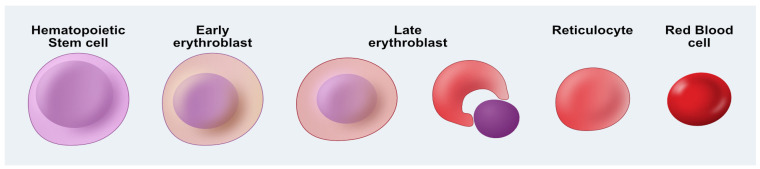
Illustration depicting the various stages of erythropoiesis from a hematopoietic stem cell to a mature red blood cell. The progression toward red color reflects increasing expression of hemoglobin throughout differentiation.

Erythropoiesis is a complex and highly regulated process, beginning with a multipotent stem cell and resulting in a enucleated, mature erythrocyte as shown in Figure 1 [43]. Briefly, the first step involves the differentiation of HSCs into more committed erythroid progenitor cells referred to as early erythroblasts. Hallmark expression of hemoglobin in high concentration begins at this phase. This is followed by the late erythroblast stage, during which the nucleus undergoes chromatin condensation, polarization toward the cell membrane, and finally extrusion out of the cell [43,44]. The resulting reticulocyte stage cells reduce their plasma membrane by roughly 20% and eject the remaining organelle components not necessary for mature RBC function [45].

In addition to the strategy based on isolation, stimulation, and differentiation of donated human HSCs from peripheral blood (PB) and umbilical cord blood (UCB), researchers are also investigating the use of immortalized HSCs [13,46] as a starting point in the manufacture of RBCs. Immortalization is a complex, multi-step process where cells are genetically manipulated such that the modified cells maintain pluripotency and differentiation ability yet are immortalized [47]. The potential benefits of an immortalized RBC precursor are tremendous. Such a cell population can be banked in large numbers without the need to continuously identify a source of human donor material (e.g., human UCB). Furthermore, homogeneous starting cell populations ensure that the differentiation process will be highly repeatable, which is critical to attaining FDA approval [48]. Lastly, the fact that mature RBCs lack a nucleus ensures that genetically manipulated donor cell DNA will not be present in the final, mature RBC which could be safely transfused into the patient. One method to achieve immortalization is to transduce HSC with an E6/E7 expression system [49]. HSCs transformed by E6/E7 expressed erythroid surface antigens, produced hemoglobin, demonstrated the ability to enucleate, and delivered oxygen comparable to adult human RBCs [49,50]. Adult-induced pluripotent stem cells (iPSCs) have also been considered as a source for mature RBCs [51,52,53]. A core group of four pluripotency-inducing genes, Oct4, Klf4, c-Myc, and Sox2, with the addition of silenced p53, promoted erythroblast proliferation while preserving their immature phenotype [49,50]. Another group demonstrating erythroid differentiation and maturation from adult fibroblasts were induced to pluripotency through overexpression of NANOG, Sox2, Lin28, and Oct4 [52]. Another potential gene of interest that has had moderate success in generating mature red blood cells is Klf1; activation of Klf1 at various stages of RBC differentiation enhanced RBC commitment and improved enucleation [53].

Regardless of the stem cell source used to generate RBCs, a major hurdle in the process is the high number of cells (2 × 10^12^) required for one patient transfusion [49,54,55]. A significant obstacle in generating large numbers of manufactured RBCs (mRBCs) is achieving high levels of enucleation (Figure 1). However, enucleation efficiency remains a challenge that must be overcome to generate large quantities of functional and manufactured mature red blood cells to become a viable alternative for patients [13,56]. Nonetheless, several groups have reported that various protocols result in close to 100 percent enucleation. Therefore, extensive studies have focused on the maximization of both cell expansion and subsequent enucleation. Multi-media step feeder protocols ranging from a 2-step to 4-step process yield between 8 × 10^4^ cells with 64% enucleation to 4.38 × 10^6^ cells with 99.4% enucleation [49,57]. In addition, it is necessary to have highly controlled processes and systems to facilitate scale-up growth and differentiation to manufacture enough RBCs to increase cost efficiency and yields.

**Figure 2:**
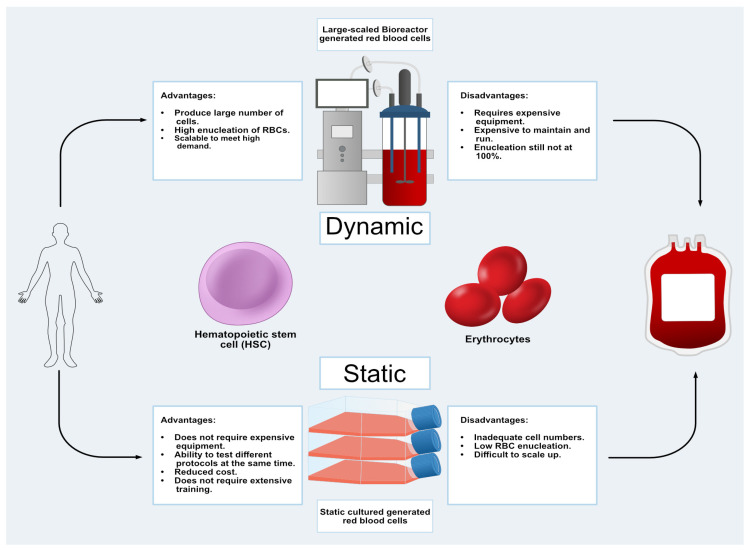
Schematic representation demonstrates the advantages and disadvantages of utilizing commercial bioreactors vs. traditional cell culturing techniques. Bioreactors serve to increase cell quantity and enucleation compared with traditional cell culture techniques, which make manufactured red blood cells a good candidate for augmenting the blood supply.

Researchers are turning to the use of bioreactor systems to recapitulate some features of RBC development in scalable, high-density in vitro culture formats [58,59,60]. Bioreactors designed for clinical applications can provide an environment in which to develop reliable and efficient cell seeding and feeding strategies that can result in a homogenous population of the desired cell type. Bioreactors should also provide mechanical environments to simulate the normal physiological conditions that cells experience in the body. A major challenge facing RBC manufacturing as a viable clinical tool is improving scale-up RBC differentiation. RBC production is a balance between maintaining optimal cell densities for growth while maximizing cell enucleation. The production of mature RBCs under experimental bioreactor conditions generates a 50-fold increase in cell expansion [58,60]. HSCs subjected to numerous growth factors and hormones such as insulin, SCF, EPO, Transferrin, and IL-3 have resulted in higher number of mature RBCs with nucleation rates of around 80% [58]. Other bioreactors that have been developed include various physiological conditions such as rocking, rotating, and fluidized bed bioreactors [60]. Other important factors that must be considered in bioreactor-based RBC manufacturing are the dissolved oxygen concentration and cell suspension density. Cells that are too densely seeded fail to proliferate well and have poor enucleation rates [60]. Although bioreactors do offer promising results and demonstrate scalable options to generate the cells required for clinical applications, the cost of these protocols far exceeds that of donated blood. Current estimates suggest that manufactured RBCs generated in bioreactors cost between 8000 and 15,000 US dollars, while donated blood costs around 240 US dollars per unit of blood; however, the cost increases significantly in patient care where the cost can reach as high as 550 dollars per unit [58,61,62]. Therefore, additional research must be conducted to enhance RBC production while reducing the overall cost of development and implementation of bioreactor-based processes to generate RBCs (Figure 2).

## 5. Synthetic Blood Products

The term “artificial blood” describes bioengineered products which can be utilized as substitutes for donated human blood [63]. For the treatment of hemorrhagic shock, artificial blood has typically been designed only for the delivery of oxygen and removal of carbon dioxide from the body [64]. The idea of artificial blood was brought to the forefront in response to the HIV epidemic during the 1980s due to the risk of infection from traditional blood transfusions [65]. There are numerous methodologies by which investigators are developing artificial blood products, including synthetic products, chemical isolation, and modern recombinant DNA technology [66].

Perfluoro-Chemicals (PFCs) are inert, colorless, nontoxic liquids with the capacity to carry oxygen [67]. PFCs can be utilized as a blood substitute for both expanding plasma volume and oxygen-carrying capacity for traumatic and hemorrhagic shocks in both civilian and military [68,69]. PFCs are not metabolized due to the strong carbon–fluorine bonds [70]. PFCs have been proposed as a blood substitute due in part to their ability to dissolve oxygen at higher rates compared to water (20×) and about twice as that of blood plasma [71]. Another unique property of PFC is heat resistance. It has been demonstrated that PFCs can withstand temperatures as high as 300 °C with any damage, making PFCs ideal for heat-based sterilization processes prior to clinical administration [72]. PFCs can also traverse very small spaces which could prove valuable in cardiovascular conditions that limit RBC passage [67].

Several PFCs have made it to clinical trials of various phases in the USA and around the world. At present, Sanguine Corp (Pasadena, CA, USA) developed PHER-O_2_ which is currently undergoing clinical trials for the treatment of stroke and myocardial ischemia [73]. Currently, Perftoran (Russian Academy) is approved in Russia and Mexico and has been administered to over 35,000 patients for the treatment of hemorrhagic shock, vascular gas embolism, and ischemia [74]. Although PFCs may be an attractive alternative to blood, there are several issues that require further investigation before they can safely be administered to human patients. PFCs are not soluble in water and therefore must be combined with emulsifiers (fatty lipids) that suspend these compounds in the bloodstream [75]. The lower oxygen binding compared with hemoglobin-based products necessitate that large amounts of PFCs are needed to transfuse in a single patient, and this could impede microcirculation as higher concentrations increase viscosity [63,68]. Their use was discontinued in the USA due to side effects such as increased stroke, cost, and immunogenicity [76]. These caveats must be addressed before PFCs can be cleared more widely for the market.

Hemoglobin-based oxygen carriers (HBOC) utilize naturally sourced Hb to emulate oxygen binding properties of RBCs [77]. HBOCs that have Hb encapsulated within artificial membrane-bound micro/nanoparticles are known as cellular HBOCs, while acellular HBOCs are composed of chemically modified Hb molecules to improve oxygen delivery and reduce toxicity [77,78,79,80]. The Hb used in HBOCs is typically isolated from either human or bovine red blood cells and is processed through sterile filtration and chromatographic techniques [79,81]. Advantages of utilizing animal-sourced Hb include reduced heme degradation, abundant source material, and using chloride ions in place of 2,3-DPG [82].

One of the advantages of using HBOCs as a blood alternative is low immunogenicity. Lacking traditional RBC antigens such as the ABO blood group can allow HBOCs to be used as a universal donor source for transfusion. Cellular HBOCs are attractive candidates due to their ability to deliver and transport oxygen, while showing reduced incidence of side effects such as high blood pressure and toxicity from free Hb in the blood [83]. HBOCs make great candidates for operational medicine in that they are amenable to long term storage [84], where encapsulation allows for a longer shelf life compared to acellular HBOCs [77]. Data suggest that lyophilized extracellular mega-hemoglobin stored in refrigerated conditions supplemented with nitrogen had a 50% reduction in oxidation while storage at room temperature lowered oxidation levels by 60%; refrigeration also preserved structural stability [84].

There are numerous acellular HBOCs that are in various stages of clinical trials; the leading candidates include conjugated, cross-linked, and polymerized HBOCs [67]. Cross-linked HBOC are generated by covalently bonding globin chains to allow them to stay attached to each other [85]. One specific cross-linked HBOC, Diasprin (Baxter, Deerfield, IL, USA), is a hemoglobin-based molecule that is cross-linked between the alpha chains [86]. Initial studies demonstrated the effectiveness of cross-linked HBOCs in cardiopulmonary resuscitation, anemia studies, and in transfusion experiments, and cross-linked HBOCs have not triggered any allogeneic response in a pig model [67]. Polymerized HBOCs are composed of 4 to 5 hemoglobin molecules that have been intermolecularly cross-linked to increase their overall size to closely resemble that of RBCs [87]. Research has led to the development of numerous polymerized HBOCs which are in various stages of clinical trials [67]. For example, a bovine based HBOC (Hemopure, Hemoglobin Oxygen Therapeutics LLC, Souderton, PA, USA) is available in the US under the FDA’s Expanded Access Program [88]. Other cross-linked HBOC products include PolyHeme (Northfield Laboratories, Evanston, IL, USA) and Oxyglobin (Hemoglobin Oxygen Therapeutics). Various studies revealed that cross-linking Hb with carbonic anhydrase (CA), superoxide dismutase (SOD), and catalase (CAT) performed superiorly when compared to HBOCs alone in a mouse model and that cross-linked HBOCs have improved storage stability in a dried state which would be advantages in events such as natural disasters and other emergency needs [89]. Conjugated HBOCs are a subclass of acellular HBOCs where inert polymers are connected on the surfaces of Hb subunits [90]. Several molecules have been developed and are in clinical trials (for example: Hemospan, Sangart Inc., San Diego, CA, USA) [72]. Conjugated HBOCs have several advantages including low immunogenicity, low toxicity, effective oxygen delivery properties, and with certain modifications, longer half-life in circulation [90,91].

HBOC investigations have been carried out since the first half of the 20th century. Although HBOCs exhibit many positive attributes as described above, their development has been troubled by drawbacks. Early studies demonstrated that a Hb cell-free system provided some therapeutic benefits, but was hampered by complications including cardiovascular and renal toxicity [77]. Hb isolated from bovine sources, although relatively cheap and abundant, risks contamination with infectious agents such as the prions that cause bovine spongiform encephalopathy [92]. One critical issue facing encapsulated Hb systems is the rapid clearance of circulation by macrophages, thus reducing their effectiveness [93]. Another challenge for HBOCs involves the lack of biochemical components in native RBCs to convert oxidized Fe^3+^ back to Fe^2+^ [67]. This inability to restore Fe^2+^ limits the oxygen binding affinity of HBOCs and thus diminishes the transport and delivery of oxygen [94]. Although the HBOCs PolyHeme and Oxyglobin demonstrated some promising results, severe side effects such as hypertension and toxicity were identified [72], and neither is currently commercially available.

Second generation HBOCs were developed because of the side-effects observed in first generation HBOCs as described above [95]. Recently, Hemarina (Morlaix, FR) developed a second generation HBOC (HemO_2_Life) which was approved by the European Union in 2022 to be utilized to perfuse kidneys prior to transplantation [96]. Research continues in the development of HBOCs which are larger in size to prevent protein denaturation and the ability to contain higher concentration of Hb [88].

## 6. Future Prospects: Opportunities for Blood Bioengineering

Transfusable blood will continue to be vital as demand for it will only increase. Manufactured blood research will need to overcome limitations that have been encountered. Induced pluripotent stem cells, with their unlimited proliferation capacity, have the ability to produce clinical-scale quantities of cells. However, the protocols to differentiate to progenitor intermediates and mature RBCs would need to be updated to perform at that scale as well. Generating cell lines from erythroblastic disorders, such as polycythemia vera [97], could also produce clinically relevant quantities of cells that are primed at an RBC precursor state. The key hindrance to any mRBC process utilizing immortal cells is enucleation [13,56]; a high rate of intrinsic enucleation and/or elimination of nucleated contaminants will be mandatory to ensure a safe, transfusable product. A recent review by Menon et al. [98] outlined molecular events that correlate with enucleation, but underscored that the precise order of necessary events needs to be determined.

Another area of active interest is reducing the production cost of mRBCs. In 2009, Timmins et al. [99] analyzed the cost of mRBC cultures with an emphasis on the culture medium. The limiting factors were recombinant technology-derived proteins such as cytokines (e.g., Interleukin-3) and morphogens (e.g., erythropoietin). The cost of creating “liquid gold” cell culture medium [99] has only increased over time due to inflation. New scale production methods for these proteins, such as transgenic tobacco plant agriculture [100,101], have the potential to address the high unit cost of cytokines.

Up to today, blood manufacturing has focused on maximizing the number of mature RBCs. Less effort has been devoted to the blood antigen identity of the potential products. Manufacturing a “universal donor” product would be the ideal situation. Mature RBCs can be extrinsically modified with enzymes that cleave the polysaccharide chains on antigenic glycoproteins [102]. Much effort has been dedicated to this process, but with limited success. If mRBCs are to be created from immortalized cells, it may be easier to use gene editing technology in the cell source to prevent blood antigens from becoming expressed. Many recently developed genetic tools [103] have the potential to modify the antigen sequences, such as ABO and Kidd on the urea transporter HUT11 [104], or to modify expression of the enzymes that synthesize antigenic glycosylations [102].

Currently, researchers are focused on recapitulating the bone marrow microenvironment to better understand erythropoiesis to identify novel signaling regulators which may be utilized to enhance the effectiveness of RBCs generated in bioreactors [105,106,107]. The bone marrow is a complex organ that consists of numerous supporting cells which include reticular cells, osteocytes, and adipocytes, and are critical for generating the biochemical signaling interactions such as growth factors and cytokines that provide the optimal environment to generate RBCs [105,106]. Previous studies demonstrated that hematopoietic cells were successfully cultivated in vivo, thus allowing researchers the potential to establish three-dimensional culture systems (3-D models) to develop a “functional” ex vivo bone marrow [107]. These 3-D models will be composed of HSCs as well as supporting cells (i.e., adipocytes, osteocytes, etc.), which will be placed along with a 3-D-printed scaffold within these 3-D culture systems to mimic the bone architecture which will allow for the formation of cell populations niches thought to be vital for the generation of RBCs [108,109]. The development of an apparatus which generates the correct environmental cues to generate RBCs will not only enhance our knowledge of hematopoiesis, but also potentially identify novel regulatory pathways not well understood which may aid in improving the current limitations encountered with manufacturing RBCs in bioreactors.

## 7. Summary

The demand for blood, whether it be for routine hospital use, natural disasters, or resuscitation of a trauma patient with massive bleeding, will likely increase in the future considering the aging donor population and increasing number of exclusions for donor qualification in the United States. Researchers have made great strides in developing blood alternatives to augment the blood supply and meet those demands. While traditional blood donations are the standard and will continue to supply the general population, alternative blood sources such as walking blood banks are good for acute shortages but probably not practical for large-scale disasters. Bioengineering offers a variety of novel solutions, notably the manufacture of both cellular and acellular blood products.

The ability to transport oxygen remains the key functional objective of current biomanufactured solutions for operation medicine. One of the key issues with manufacturing blood, as highlighted with mRBCs, is the production scale-up and the related costs needed to meet practical clinical demands as well as quality standards such as high enucleation percentage for economic feasibility of a commercial mature mRBC product moving forward. Nonetheless, several groups have reported that some protocols result in close to 100 percent enucleation but whether this can be achieved at high volumes economically remains to be investigated.

Synthetic blood products solutions have also been bioengineered and, as with mRBCs, are under development exclusively for the ability to deliver oxygen to critical organs. Synthetic blood alternatives such as HBOCs have several advantages to traditional blood and perhaps mRBCs; HBOCs reduce the immunogenicity of blood, have a longer shelf life, and can be lyophilized (freeze-dried) and reconstituted in areas where it is needed. This latter point can be significant as mRBCs, like donated blood, need to be stored and stockpiled long term, requiring cryopreservation with cold chain transportation up to point of need. Costs for sustaining a cold chain transportation network are costly and in austere environments, it is often very challenging to ensure the medicine remains operational. Following an earthquake or hurricane, there is typically no electricity to support refrigerated blood supplies. The lack of a refrigeration requirement for HBOCs significantly simplifies logistical concerns over storage and shipping for use in austere environments where traditional blood storage facilities may not be available or rendered non-operational in inclement conditions, such as after a natural disaster or in a combat zone.

Although we still have a long way to go, the development of alternatives to traditional blood donations is needed to ensure sustainment of the national blood supply. Bioengineered solutions of mRBCs and synthetic products like HBOCs have shown immense promise. Improvements in bioreactor design and biomanufacturing may provide key innovations for improving the cost efficiencies for production and must continue to be investigated to meet the urgency of the anticipated growing blood shortage in the United States [15].

## Figures and Tables

**Table 1:** Social and medical disqualifications that prevent people from donating blood.

Ineligibility for Blood Donations		
	Drug users who used needles to take drugs.	
Social Disqualifications	Engaged in sex for money.	
	Recent tattoos and piercing work.	
	HIV-1	HIV-2
	Hepatitis-B	Hepatitis-C
	Chagas disease	Babesiosis
Medical Disqualifications	Psoriasis	Creutzfeldt-Jakob disease
	HTLV-1	HTLV-2
	Syphilis	Herpes (HSV)
	Lymphoma, leukemia	Anemia

## Abbreviations

The following abbreviations are used in this manuscript:

RBC	Red blood cell
WBB	Walking blood bank
HSC	Hematopoietic stem cell
HBOC	Hemoglobin based oxygen carrier
PFC	Perfluoro-Chemicals
IPSCs	Induced Pluripotent Stem Cells
UCB	Umbilical cord blood
SCF	Stem cell factor
Hb	Hemoglobin
IL-3	Interleukin-3
EPO	Erythropoietin
2,3-DPG	2,3-diphosphoglycerate
Oct4	Octamer-binding transcription factor 4
KLF1	Krüppel-like factor 1
Sox2	SRY-box 2
NANOG	Nanog homeobox
